# Revisiting the effect of PCR replication and sequencing depth on biodiversity metrics in environmental DNA metabarcoding

**DOI:** 10.1002/ece3.8239

**Published:** 2021-10-22

**Authors:** Sabrina Shirazi, Rachel S. Meyer, Beth Shapiro

**Affiliations:** ^1^ Department of Ecology and Evolutionary Biology University of California Santa Cruz Santa Cruz California USA; ^2^ Howard Hughes Medical Institute University of California Santa Cruz Santa Cruz California USA

**Keywords:** alpha diversity, beta diversity, eDNA, methods, PCR replicates, sedaDNA, sequencing depth

## Abstract

Environmental DNA (eDNA) metabarcoding is an increasingly popular tool for measuring and cataloguing biodiversity. Because the environments and substrates in which DNA is preserved differ considerably, eDNA research often requires bespoke approaches to generating eDNA data. Here, we explore how two experimental choices in eDNA study design—the number of PCR replicates and the depth of sequencing of PCR replicates—influence the composition and consistency of taxa recovered from eDNA extracts. We perform 24 PCR replicates from each of six soil samples using two of the most common metabarcodes for Fungi and Viridiplantae (ITS1 and ITS2), and sequence each replicate to an average depth of ~84,000 reads. We find that PCR replicates are broadly consistent in composition and relative abundance of dominant taxa, but that low abundance taxa are often unique to one or a few PCR replicates. Taxa observed in one out of 24 PCR replicates make up 21–29% of the total taxa detected. We also observe that sequencing depth or rarefaction influences alpha diversity and beta diversity estimates. Read sampling depth influences local contribution to beta diversity, placement in ordinations, and beta dispersion in ordinations. Our results suggest that, because common taxa drive some alpha diversity estimates, few PCR replicates and low read sampling depths may be sufficient for many biological applications of eDNA metabarcoding. However, because rare taxa are recovered stochastically, eDNA metabarcoding may never fully recover the true amplifiable alpha diversity in an eDNA extract. Rare taxa drive PCR replicate outliers of alpha and beta diversity and lead to dispersion differences at different read sampling depths. We conclude that researchers should consider the complexity and unevenness of a community when choosing analytical approaches, read sampling depths, and filtering thresholds to arrive at stable estimates.

## Graphical abstract

Previous studies examining the influence of methodological choices in environmental DNA processing on the resulting observed communities are a great resource in designing methods of a study, and accurately interpreting results. Here, we designed a methodological experiment to address the influences of PCR replication, sequencing depth, and a minimum read threshold on measures of biodiversity. We found stability between PCR replicates in high abundance taxa, and high variation in low abundance taxa, which significantly influence alpha and beta diversity.

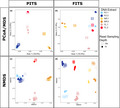

## 1 INTRODUCTION

Environmental DNA (eDNA) metabarcoding is gaining traction as a biomonitoring tool (e.g., Deiner et al., 2021; Mejia et al., 2021) and for testing hypotheses about biotic and abiotic drivers of changes in community composition (Deveautour et al., 2018; Erlandson et al., 2018). Metabarcoding is used to measure species richness and compositional turnover in environmental samples, including changes in biodiversity over time (Bálint et al., 2018; Epp et al., 2015; Willerslev et al., 2014) and across large geographic ranges (e.g., the sunlit ocean, de Vargas et al., 2015; human impact gradients, DiBattista et al., 2020). This work has led to development of new essential biodiversity variables (Jetz et al., 2019) and bioindicators of environmental change (Kissling et al., 2018; Lin et al., 2021). In addition, because metabarcoding can be performed simultaneously for multiple loci that target different taxonomic groups, the technique can be used in applied ecology and habitat management without *a priori* knowledge of community composition. Despite the potential of metabarcoding, however, variation in metabarcoding results among biological samples from the same location, and even among technical replicates from the same DNA extract, continues to complicate eDNA experimental design at all stages of sample collection, processing, and data analysis (Beng & Corlett, 2020).

Variation among replicates of metabarcoding experiments arises due to a combination of biological and technical biases. Biological biases reflect differences among taxa in the probability of DNA preservation due, for example, to organism size, seasonality, and behavior (Beng & Corlett, 2020). Technical biases are introduced by experimental choices during field sampling, data generation, and bioinformatic analysis. For example, technical biases can be introduced if DNA isolation protocols (Deiner et al., 2018; Dopheide et al., 2019), PCR polymerases (Nichols et al., 2017), and metabarcoding primers (Alberdi et al., 2017; Clarke et al., 2014; Deagle et al., 2014) preferentially recover taxa with particular physiological traits or genetic sequences. Biases may also emerge if taxonomic profiles become skewed during PCR due to PCR runaway (Polz & Cavanaugh, 1998), tag jumping (Taberlet et al., 2018), and overamplification (McPherson & Moller, 2006), although the latter can be mitigated somewhat by using quantitative PCR (qPCR) to determine the most appropriate number of PCR cycles (Murray et al., 2015). Finally, biases can be introduced by the stochastic nature of PCR amplification (Beentjes et al., 2019; Leray & Knowlton, 2017), such that taxa that are rare in the DNA extract may become common in the postamplification pool if amplified during an early PCR cycle (Nichols et al., 2017). As a consequence of these combinations of biases, replicate metabarcoding PCRs can provide significantly different taxonomic profiles, and these profiles will always be limited to *amplifiable* molecules and not, therefore, represent the true biodiversity at a given site.

Previous studies have explored some of the reasons why PCR replicates often have different taxonomic profiles. A goal of this work has been to make generalizable recommendations as to how best to avoid or mitigate these potential biases, which has proven difficult. Smith and Peay (2014), for example, reported that two of the most common measures of biodiversity—alpha and beta diversity—did not change with higher numbers of PCR replicates. However, this study sequenced pooled rather than individual replicates, such that fewer reads were sampled from each replicate as the number of replicates increased, which may affect recovery of rare taxa. In a landmark study, Ficetola et al. (2015) used species occupancy modeling to determine the most appropriate number of PCR replicates based on predicted taxon abundance. In contrast to Smith and Peay (2014), this study found that as many as eight replicates should be used if the probability of detection of rare taxa was not high. When they tested this hypothesis using biological samples, they confirmed that using more replicates increased observance of rare taxa and recommended bespoke replication strategies based on biological information. However, their replicate design included PCRs from multiple extracts rather than a single extract and so did not explicitly address differences between true replicates.

Although Ficetola et al. (2015) found that higher numbers of replicates were often important in surveying biodiversity, few studies use high replication to date. Nonetheless, studies have continued to show the importance of replication in surveying diversity. Alberdi et al. (2017), Leray and Knowlton (2017), and Beentjes et al. (2019) each performed three replicate PCRs and found that alpha diversity increased as replicates were added, suggesting that replication recovers rarer taxa. To test this explicitly, Dopheide et al. (2019) performed up to 10 replicate PCRs for each of four metabarcodes and estimated species accumulation curves as PCR replicates were added. They found that curves began to flatten only after this relatively higher level of replication and predicted that species accumulation would plateau with 10–20 replicates. More recently, Rojahn et al. (2021), while exploring the effect of PCR replication for detecting rare species of fish, suggested that high PCR replication is not sufficient in some cases to detect rare taxa, in particular where common taxa may swamp the PCR and inhibit detection.

The influence of replication when surveying alpha diversity is better understood than is its influence on beta diversity—a measure of dissimilarity between sites or samples. Smith and Peay (2014), for example, observed no influence of the number of pooled replicates on beta diversity when sequencing depth was held constant, although their pooled sequencing strategy may have reduced the possibility that rare taxa would be observed. Beentjes et al. (2019) and Hajibabaei et al. (2019) did not pool replicates and therefore probably recovered more rare taxa given their read sampling depth, but also found little to no effect of replication on beta diversity. Instead, Beentjes et al.(2019) found that including biological replicates sampled across space and over time was more likely than PCR replication to affect beta diversity, probably because increasing the number of biological replicates samples additional taxa.

PCR replication is not the only experimental choice that can influence recovery of rare taxa within a DNA extract, and therefore affect measures of alpha and beta diversity. Sequencing read depth, or the number of mapped reads to which each PCR amplicon is sequenced, may also affect the probability that rare taxa are observed. To test explicitly the influence of sequencing depth, Alberdi et al. (2017) compared read depths of ~2500 to ~25,000 reads per replicate and found that alpha diversity increased with sequencing depth. Smith and Peay (2014) calculated pseudobeta diversity among replicates generated by pooling different numbers of PCR replicates before sequencing and calculating diversity of the pooled samples at different rarefied depths. They found that dissimilarity between replicates decreased with increased sequencing depth and concluded that sampling depth was more important than replication when recovering biodiversity within a PCR amplicon pool. While these results suggest that surveyed biodiversity increases with sequencing depth, how sequencing depth influences beta diversity remains underexplored.

Here, we examine how two experimental choices—number of PCR replicates and depth of sequencing for each replicate—affect the composition and consistency of diversity estimates in metabarcoding experiments. Because metabarcoding can only recover taxa that are present in and amplifiable from a DNA sample, we are not addressing the effect of these variables on recovering the complete *biological* diversity of a particular site. Instead, our goal is to provide new insights into the reliability and replicability of PCR to recover the diversity of *amplifiable* taxa. We prepare a total of six DNA extracts from three geographic locations with distinctive biodiversity profiles, and, following the conclusions of Dopheide et al. (2019), perform 24 individually barcoded replicate PCRs from each extract. We sequence each PCR replicate to a target depth of >50,000 reads and calculate alpha and beta diversity of replicates. To explore differences in potential bias between taxonomic groups, we perform this experiment with two commonly used metabarcodes that capture different phylogenetic biodiversity: the *Internal Transcribed Spacer* (*ITS*) for Fungi (*ITS1*) and for Viridiplantae (land plants and algae; *ITS2*). We use standard statistical approaches to explore how PCR replication and read sampling depth influence metabarcoding‐based biodiversity estimates, and address explicitly detection of rare taxa and inference of community composition.

## 2 METHODS

### 2.1 Soil collection

We collected two soil samples from three ecologically distinct locations for a total of six samples. Two were from St Paul Island, Alaska, USA (StP.1: arctic tundra along wetlands, 57.136074, −170.82537; StP.2: arctic tundra, 57.10577, −170.10563) and four were from sites in California, USA: two from Fort Ord Natural Reserve in Marina (FO.1, an open sand dune: 36.68448, −121.77731; FO.2, a chaparral ecosystem: 36.68301, −121.78071), and two from Younger Lagoon in Santa Cruz (YL.1, the basin of a coastal lagoon: 36.950081, −122.066756; YL.2, a grassland coastal terrace: 36.949314, −122.063575).

We designed field sampling protocols to minimize risk of cross‐contamination. At each site, we wore clean gloves and used a trowel sterilized between samples to collect soil from 2″ to 6″ below the surface in 50‐ml falcon tubes.

### 2.2 DNA extraction, amplification, sequencing, and taxonomy assignment

We processed each soil sample in the UCSC Paleogenomics Lab eDNA room where no PCR amplification occurs, following clean room protocols. We homogenized and removed large plant matter (leaves and roots) from each sample, and subsampled two 0.25 g aliquots of sediment from each sample. We extracted DNA from each of the 12 samples using the Qiagen PowerSoil kit and protocol (Qiagen), including one negative extraction control without soil. We pooled the duplicate extracts for each site to ensure that enough DNA extract was available for the replication experiments.

We performed metabarcoding on each of the six extracts using the *ITS2* region targeting plants (which we abbreviate as PITS) and the *ITS1* region targeting fungi (FITS). We chose barcodes that were (1) among the most commonly used plant (Ankenbrand et al., 2015) and fungal (Nilsson et al., 2018) metabarcodes in eDNA; and (2) unlike other common barcodes that can only identify taxa to higher taxonomic levels, these barcodes can identify taxa to genus and sometimes species and are therefore less prone to lumping reads from different species into a single low‐resolution taxon named only at the family or order level. For PITS, we used primers described by Yao et al. (2010); ITS‐S2F and ITS‐S3R, and for FITS, we used primers from White et al. (1990); ITS5‐forward and Epp et al. (2012); 5.8S_fungi‐reverse. The expected amplicon length was 450–480 base pairs (bp) for PITS and 200–350 bp for FITS.

For each extract, we used qPCR to assess PCR inhibition and determine the appropriate number of PCR cycles for metabarcoding (Murray et al., 2015). We performed qPCR with the Qiagen Multiplex PCR Master Mix following manufacturers’ protocol with a spiked 1:2000 dilution of SYBR Green 1 Dye. In triplicate for each extract, we set up a serial dilution of 1:0, 1:1, and 1:3 extract to water proportions of the 2 µl DNA extract and compared qPCR Ct values across the dilution series. We observed no inhibition and proceeded with undiluted extracts. We determined the optimal number of PCR cycles for each extract and primer as the cycle after which the exponential amplification phase ended.

We followed a “2‐step” protocol to build amplicon sequencing libraries (Nichols et al., 2017) using the same reagent setup as for qPCR with the appropriate number of cycles and without SYBR Green. For each extract, we performed 24 replicate PCRs with PITS and 24 PCR replicates with FITS. We amplified four PITS and four FITS PCR replicates from the extraction negative control (no sediment) and added two additional PCR negative controls (no extract) for each marker. We purified amplicon pools with SPRI beads (Beckman), then indexed all PCR products individually using Kapa Hifi (Roche), following 25 μl manufacturer's protocol, to add eight‐bp dual indices, followed by a second SPRI bead clean. We used unique combinations of dual indices for each PCR replicate. We then quantified the concentration of DNA in the purified amplicon libraries with a Nanodrop (Thermo Scientific) and pooled the libraries by equimolar ratios into PITS and FITS pools. We then quantified the pools with a Qubit fluorometer (Thermo Fisher) and estimated average fragment sizes with a fragment analyzer.

To detect index swapping (incorrect index assignment between adjacent clusters; van der Valk et al., 2019) during sequencing, we amplified the PITS metabarcode from a DNA extract of spiral ginger **(** *Costus pulverulentus*), which is native to the neotropics and not found in California or Alaska. We amplified the ginger samples separately from all environmental samples, removing the possibility of cross‐contamination during amplification. We generated three replicate PCR amplicon libraries from the spiral ginger extract following the 2‐step protocol described above.

We pooled and sequenced 308 sediment and three spiral ginger libraries on an Illumina MiSeq v3 600 cycle kit for 2 × 300 bp reads. We targeted 100,000 reads per FITS library and 50,000 reads per PITS library, based on the anticipated higher taxonomic richness amplified by FITS and higher discard rate of FITS‐amplified sequences due to the incompleteness of fungal taxonomy databases.

We used the first step of the *Anacapa Toolkit* (Curd et al., 2019) to perform quality control trimming and generate merged and unmerged forward and reverse amplicon sequence variants (ASVs). We then used the second step of the *Anacapa Toolkit* (Curd et al., 2019) to cluster ASV tables into taxonomy tables, which employs a Bayesian Least Common Ancestor approach to classify taxa above statistical support cut‐offs (see full description of *Anacapa* in Text S1; Gao et al., 2017). Taxonomy is assigned in the *Anacapa* pipeline with both a local and global bowtie2 alignment of ASV clusters to CRUX databases built from NCBI nr/nt data (CRUX database generation described in Text S1).

### 2.3 Data filtration and analysis

We used the PCR and DNA extraction negative controls to detect and remove contaminants and the positive ginger control to infer the rate of index swapping. We converted taxonomy tables and the PCR replicate‐associated metadata to *phyloseq* (v. 1.22.3; McMurdie & Holmes, 2013) objects using *Ranacapa* (Kandlikar et al., 2018). We then used the R package *decontam* (v1.1.0; Davis et al., 2018) to remove identified contaminants using prevalence 0.1 between true samples and controls. To test for index hopping, we examined the species composition of spiral ginger extracts and looked for spiral ginger reads in our soil extracts.

To simulate PCR replicate diversity at different read depths, we randomly drew different numbers of reads (rarefied) from the decontaminated taxonomy tables for each DNA extract. We used the rarefy_even_depth() function of *phyloseq* to rarefy our data at depths of every thousand between 1000 and 20,000 (ex. 1k, 2k, 3k…). As we increased rarefaction depth, some libraries that were sequenced less deeply dropped out of the analysis. Following rarefaction, we generated three datasets for each rarified library in which we applied minimum read thresholds of 2, 5, and 10. We applied the minimum read threshold to each technical PCR replicate individually. The taxon richness average of 25 rarefactions per PCR replicate was plotted using data filtered with a minimum read threshold of 5.

We tested false positives in PITS data by evaluating the likelihood that taxa detected in a DNA extract are known local taxa reported to the Global Biodiversity Information Facility (GBIF.org). We used GBIF data grabs from https://doi.org/10.15468/dl.yptmrz for Younger Lagoon and Fort Ord extracts and used https://doi.org/10.15468/dl.7c8huv for St. Paul Island. We performed 1‐tailed *t*‐tests in R to compare these local survey taxa to PITS taxa.

We generated empirical and extrapolated taxon accumulation curves for datasets prior to estimating various Alpha diversity metrics using the R package *iNEXT* (Hsieh et al., 2016). We implemented *iNEXT* with *q* = 0, datatype = “abundance,” knots = 40, se = TRUE, conf = 0.95, nboot = 50 extrapolate to replicates to twice their true read sampling depth. We performed outlier tests on extrapolated observed richness by identifying points that fall outside values of 1.5 times the interquantile range. We calculated observed richness, the Shannon diversity index (Shannon, 1948), and Simpson index (Simpson, 1949) with the *vegan* package in R (Oksanen et al., 2018). While observed alpha diversity considers only taxon presence, the Shannon and Simpson's estimators consider both the relative abundance of taxa within a sample in addition to taxon presence. We then performed two‐sided *t*‐tests and chi‐square tests in R *stat*.

We performed statistical tests for beta diversity, including the local contribution to beta diversity (LCBD), using *MicrobiomeSeq* (Ssekagiri et al., 2017) in R, which draws on the *adespatial* (Dray et al., 2018) *beta*.*div* function. We analyzed community composition using unconstrained ordination calculated with the binary Jaccard dissimilarity distance in *vegan* for datasets rarefied to 1000 and 10,000 reads and with a minimum read threshold of five reads. We then computed the relative sizes of dispersion of PCR replicates per DNA extract using the *betadisper* function in *vegan* using type “median,” bias.adjust = TRUE. If groups were significantly different, we also performed ANOVA followed by Tukey HSD tests. To determine possible causes of dispersion, we used the unconstrained Random Covariance Model RC(M) with package *RCM* (Hawinkel et al., 2019) with a dataset rarefied to 5000 reads and with a minimum read threshold of five reads. RC(M) was performed for each DNA extract separately, and results were plotted to show the taxonomic vectors as arrows and PCR replicate samples as dots.

## 3 RESULTS

### 3.1 Data summary and evaluation of potential contaminants and false‐positive taxa

We generated an average of 78,809 PITS sequences (range: 9352–282,579; Table S1) and 88,987 FITS sequences (range: 15,409–382,888; Table S2) for each of our 288 amplicon libraries (24 PCR replicates for each of six extracts, two markers). Following adapter removal and quality trimming, we retained an average of 37,640 PITS reads (range: 6148–166,279; Table S1) and 63,436 FITS reads (range: 12,360–323,310; Table S2) per PCR replicate.

Based on the sequence composition of the three *Costus pulverulentus* samples, we found no evidence of index hopping between libraries during sequencing. We generated 52,675–299,400 reads for each of the three ginger samples, all of which were assigned to *Costus pulverulentus*. Additionally, not a single sequence in any environmental sample was assigned to *C*. *pulverulentus*. After prevalence‐based decontamination, which identified and removed two taxa from the FITS dataset (*Malassezia restricta* and *Stereum hirsutum*), 1099 unique taxa were retained in the FITS results and 353 were retained in the PITS results (Table S3), with 278 of the FITS taxa and 50 of the PITS taxa represented by only a single read. We assumed single read taxa and other low abundance taxa (<10 reads) were potential false positives and filtered these out with thresholds of 2, 5, and 10 reads in downstream analyses. We used traditional observation cross‐validation and an analysis of congeneric species in our results (Text S2; Table S4) and found that while some low frequency taxa may be false positives, they are not overrepresented as singleton observations compared to taxa cross‐validated as likely true, and therefore are not expected to impact downstream results. Following decontamination, most taxa were identified to the species level, although some were identified to higher taxonomic levels (PITS: 260 species, 74 genus, 19 family, 7 order, 2 class; FITS: 875 species, 178 genus, 35 family, 18 order, 8 class; see Table S3).

### 3.2 The influence of read sampling depth on alpha diversity

**FIGURE 1:**
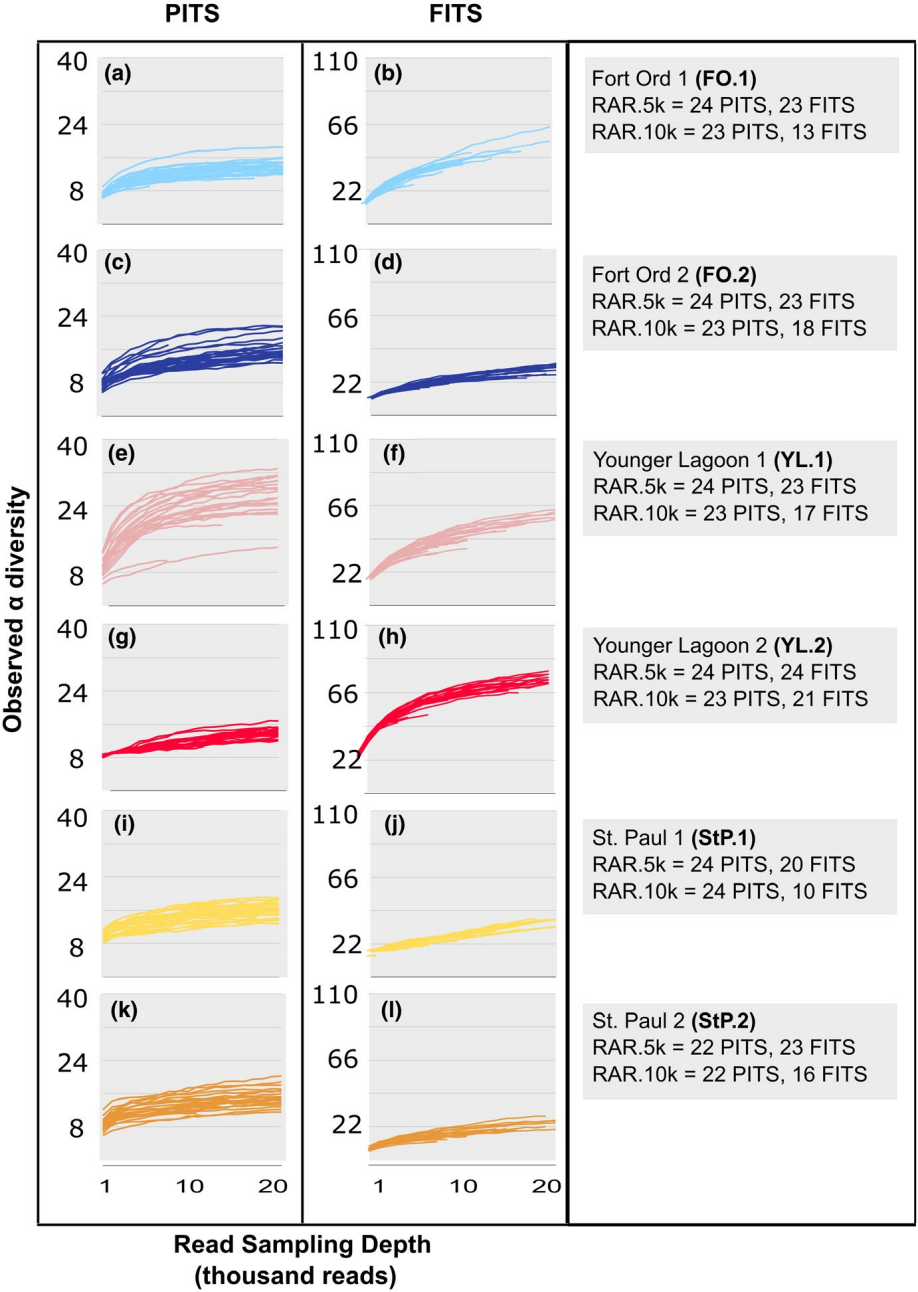
Rarefaction curves tracking observed number of taxa identified among PCR replicates as a function of read sampling depth for the PITS and FITS datasets. Plots are created from the average of 25 rarefactions at each round thousand sampling depth between 1000 and 20,000 reads, and a minimum read threshold of 5. Each line represents one PCR replicate. Termination of a line prior to the 20,000 read sampling depth denotes missing data. RAR = Retained after rarefaction, referring to the number of PCR replicates retained in analysis following rarefaction to read sampling depths of 5000 and 10,000 reads. All 24 replicates for each of the six DNA extracts, and both primers had sufficient data at 1000 reads to be included in analysis (RAR.1k = 24 for all DNA extracts and amplicons)

**FIGURE 2:**
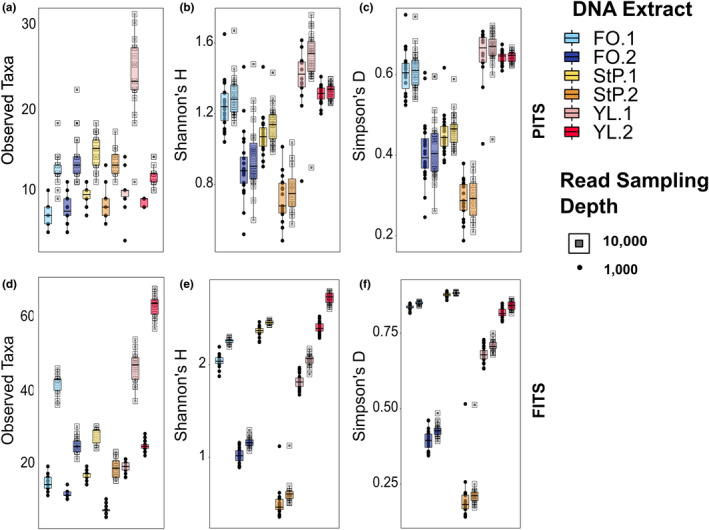
A comparison of PITS results and FITS results as observed (a and d), Shannon (b and e), and Simpson (c and f) alpha diversity measured with read sampling depths of 1000 (circles) and 10,000 (squares), and a minimum read threshold of five. PCR replicates with fewer than 10,000 reads drop out from analysis and therefore may only be plotted with 1000 reads. Each dot represents a single PCR replicate

Taxon accumulation curves (Figure 1) show that for all DNA extracts and PCR replicates, the PITS curves surpass the inflection point where slope begins to decrease (asymptote) at a sampling depth under 5000 reads, but the inflection point is less apparent in the FITS dataset. Figure 2 shows that increasing the read sampling depth from 1000 to 10,000 reads resulted in an average 1.8‐fold increase in observed alpha diversity for PITS and 2.4‐fold increase for FITS (Table S5). Shannon and Simpson diversity did not significantly increase with read sampling depth for most extracts in the PITS dataset but did significantly increase with all FITS datasets (Table S5).

We found the extrapolated variance in richness among PCR replicates of a single DNA extract was high for both metabarcodes and that the degree of variation was not consistent across extracts from different habitats. Observed richness estimates were rarely normally distributed and variance was high, with up to five replicates from the same extract being outliers from the mean, and a total of 17 PCR replicate outliers across all samples (Table S6). After outlier removal, PCR replicate richness at the extrapolated asymptote still exhibited multiple fold differences in PITS and standard deviations equivalent to up to 30% of the maximum richness of the group (Table 1). We found the highest fold differences in observed richness in the PITS dataset from YL.1 (Figures 1e and 2a), a site situated within a marine lagoon at a location that is regularly inundated with both marine water and stream runoff. We observed fewer outlier replicates in extrapolated richness for FITS, with only up to two outliers per group, but that variation was high, with standard deviations up to 21% of the maximum richness of the group (Table 1). We observed the highest fold differences in observed richness in FITS at YL.1 and FO.2.

**TABLE 1:** Variation in extrapolated taxonomic richness among PCR replicates after outlier removal

Extrapolated site richness				
SITE	MIN	AVG	MAX	STDEV
Plant *ITS2* (PITS)				
FO.1	21.2	31.3	48.3	5.8
FO.2	2.0	32.8	68.3	18.6
YL.1	2.0	63.2	122.8	31.4
YL.2	3.0	34.6	49.8	10.5
StP.1	1.0	24.6	51.4	15.2
StP.2	5.0	28.9	56.2	12.9
Fungal *ITS1* (FITS)				
FO.1	46.4	87.3	111.2	14.9
FO.2	24.5	75.6	106.7	22.2
YL.1	48.6	106.5	156.1	27.9
YL.2	97.0	149.3	199.8	24.4
StP.1	30.7	51.2	78.4	11.6
StP.2	34.2	50.6	75.2	12.0

### 3.3 PCR replicates under different read sampling depths and minimum read thresholds

**FIGURE 3:**
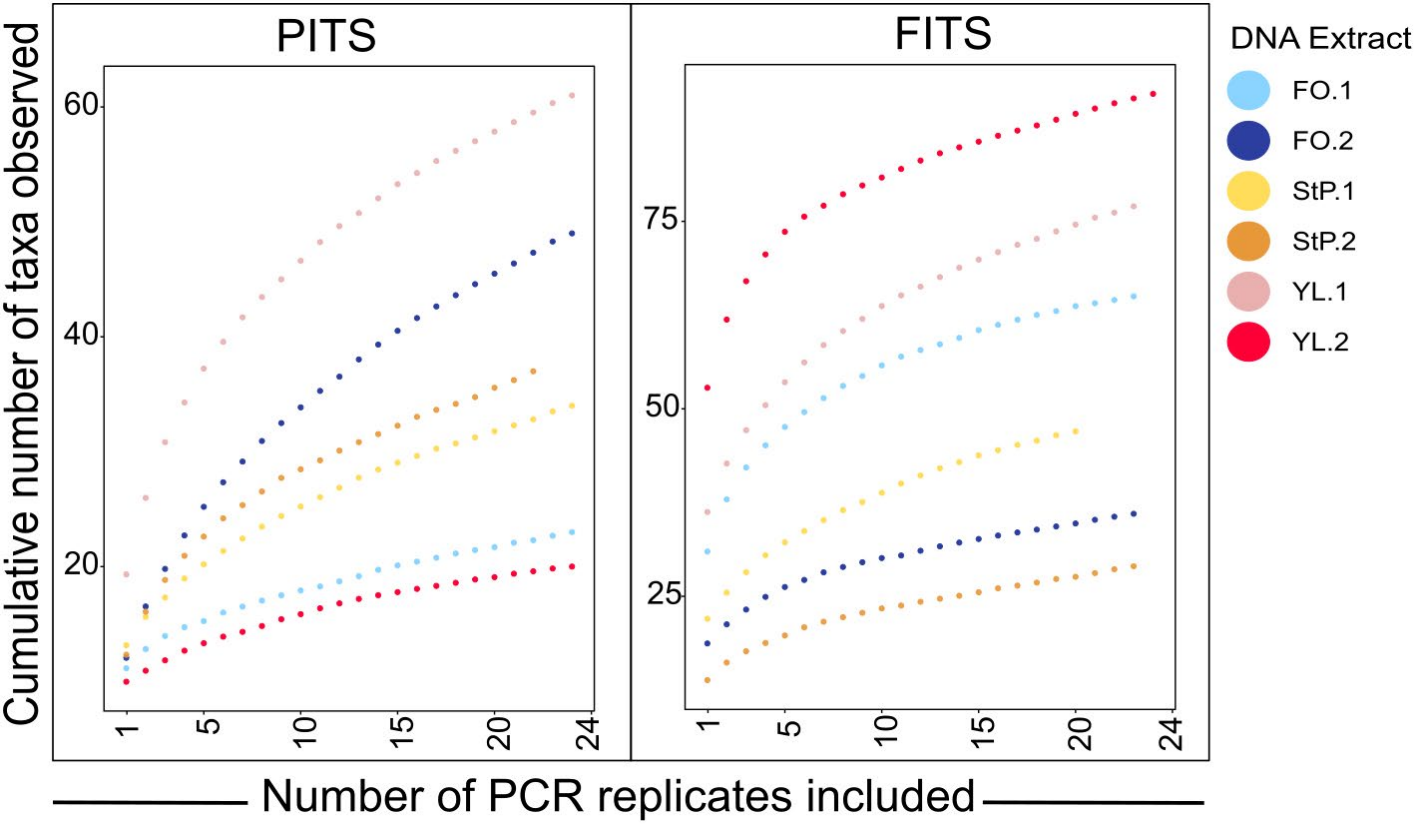
Rarefaction curves describing the cumulative number of taxa detected with increasing number of PCR replicates, each sampled to a read depth of 5000 reads and using a minimum read threshold of five. We chose to plot the rarefaction depth at 5000 reads, as the exponential increase in taxon accumulation had begun to plateau by this read depth (Figure 1) and fewer PCR replicates had to be removed compared to higher read sampling depths. Each line reflects the average of 100 bootstraps in which the order at which individual replicates were added was shuffled

To measure the presence of low abundance and possibly unique taxa, we calculated increases in alpha diversity as PCR replicates are added to a combined dataset, bootstrapping the analysis 100 times, and plotting the mean (Figure 3). Intriguingly, we did not observe a plateau in species richness even after all 24 PCR replicates were included, indicating that this relatively high number of PCR replicates was insufficient to fully sample the diversity of taxa within the DNA extract (Figure 3). Generally, increasing read sampling depth increased the number of PCR replicates needed to reach saturation, and increasing the minimum read threshold lowered the number of replicates required to reach saturation (Table 2), where we define “saturated” as when the number of taxa increases by less than one on average when another PCR replicate is added. While the curves were different for each of the DNA extracts, the trend was consistent among them.

**TABLE 2:** Number of PCR replicates required to reach saturation of taxon accumulation curve, defined as the point at which taxon accumulation curve (shown in Figure 5) increases by on average (calculated with 100 bootstraps) less than one taxon with the addition of another PCR replicate

Number of PCR replicates to reach saturation of taxon accumulation curve							
Read sampling depth	Minimum read cutoff	FO.1	FO.2	YL.1	YL.2	StP.1	StP.2
Plant *ITS2* (PITS)							
5k	2	5	20	17	4	8	10
	5	3	17	15	1	8	9
	10	2	10	12	1	3	8
10k	2	9	17	>23	5	15	19
	5	4	13	19	3	10	18
	10	3	9	16	1	9	9
Fungal *ITS1* (FITS)							
5k	2	>23	>23	>23	>24	>20	19
	5	11	7	15	12	12	6
	10	9	2	6	9	4	2
10k	2	>13	>18	>17	>21	>10	>16
	5	>13	>18	>17	>21	>10	>16
	10	>13	4	12	9	7	3

Note

“>X” denotes that greater than the maximum number of replicates X retained after rarefaction is needed to suffice this point.

**FIGURE 4:**
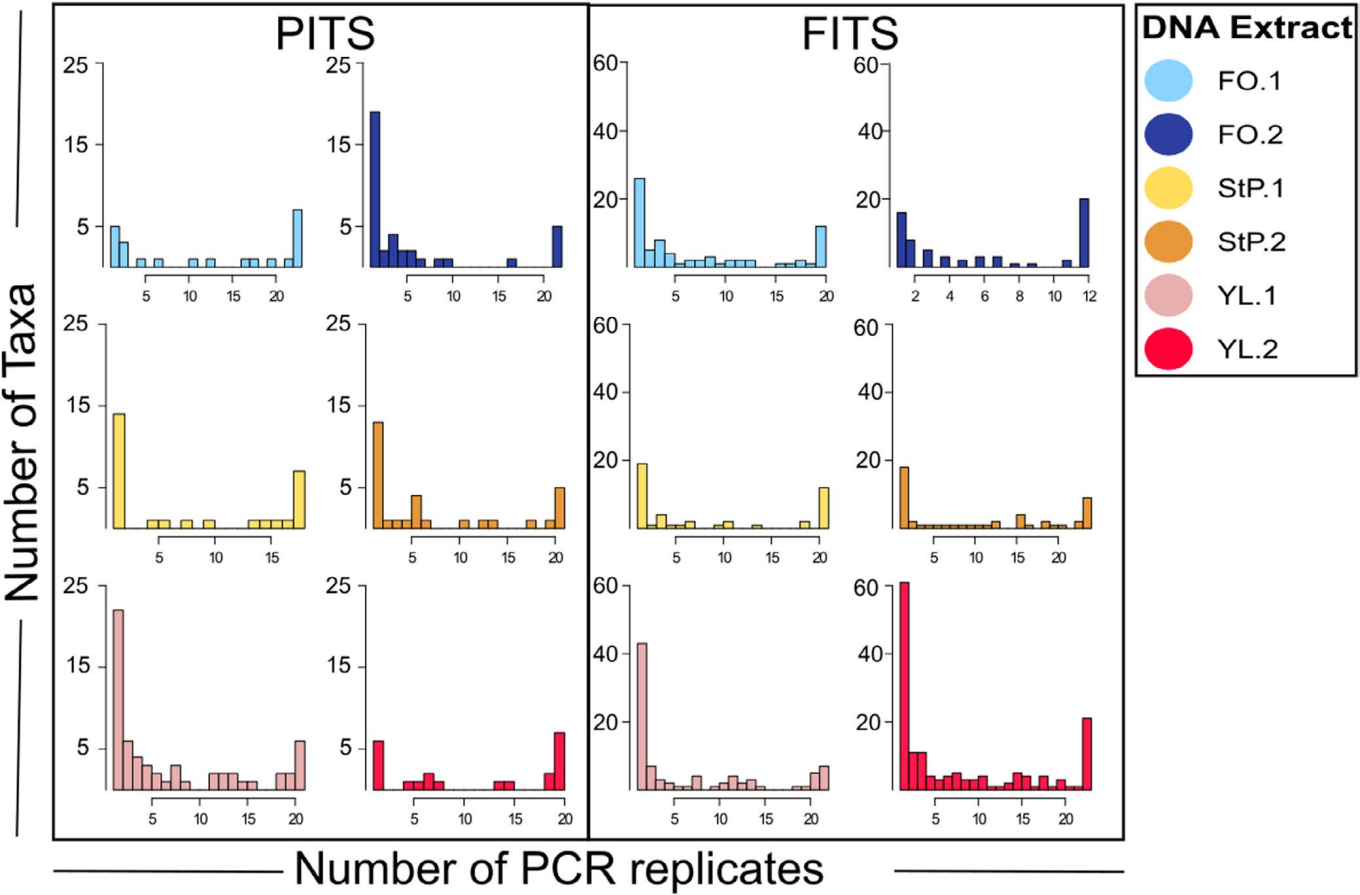
Histograms describing the frequency of individual taxa detected across PCR replicates, each sampled to a read depth of 5000 reads and using a minimum read threshold of five, out of the total 24 replicates. The right‐most bar in each plot is a count of taxa present in all replicates, while the left‐most bar is a count of taxa present in only one replicate

**FIGURE 5:**
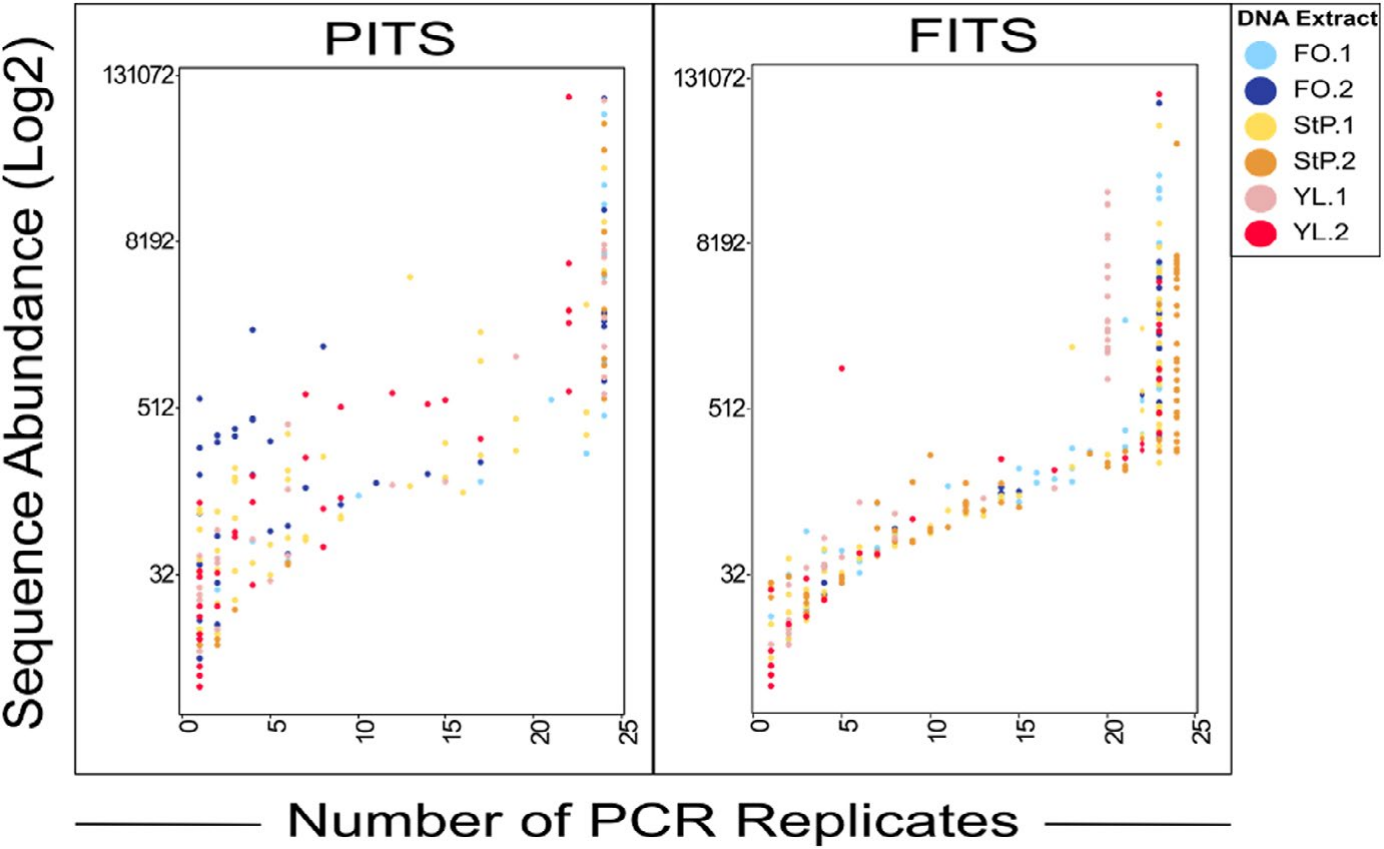
Taxon accumulation curves by PCR replicate. Each dataset comprises 5000 subsampled reads and incorporates a minimum read threshold of five. Sequence abundance is plotted as log‐transformed counts of the number of reads per PCR assigned to a particular taxon, averaged across the PCRs in which that taxon is observed. We find a significant positive correlation between the number of PCR replicates in which a taxon is observed (fitted linear model results—PITS: *p* < 2e−16, *T* = 24.73, adjusted *r* ^2^ = .7324; FITS: *p* < 2e−16, *T* = 39.91, adjusted *r* ^2^ = .8219). Each dot represents an individual taxon and is colored according to DNA extract

Most taxa were present either in only one PCR replicate or in all PCR replicates (Figure 4). We found a significant correlation between a taxon's within‐replicate sequence abundance and its frequency across replicates at all read depths and minimum read thresholds (Figure 5). Taxa present in all PCR replicates in the 5000 read dataset (Figure 5) were at sequence frequency 0.2–36.7% in the FITS dataset (average 3.22%) and sequence frequency 0.36–74.68% in the PITS dataset (average 13.58%). Taxa present in only a single PCR replicate in the 5000 read dataset (Figure 5) were at sequenced frequency up to 0.02% (average 0.006%) in the FITS dataset and up to 12% (average 0.6%) in the PITS dataset. Increasing the minimum read threshold reduced the number of taxa detected in only a single PCR replicate, while decreasing the minimum read threshold increased the number of taxa detected in a single replicate (Figures S1 and S2).

### 3.4 Composition and relative abundance (RA) variation across PCR replicates

**FIGURE 6:**
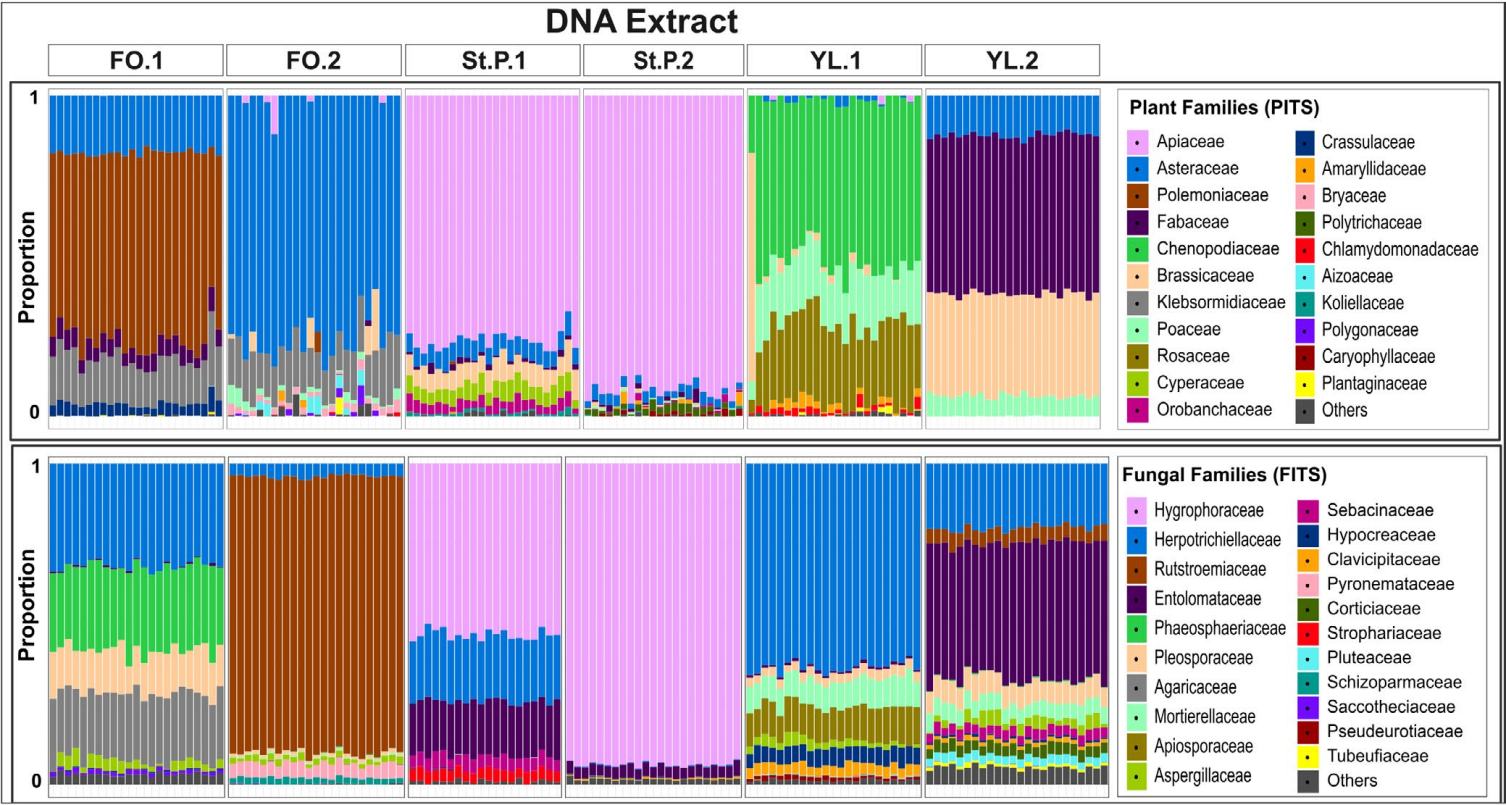
Relative abundance of plant and fungal families detected with 5000 reads and a five read minimum threshold. Each bar represents one PCR replicate. Only the 20 most abundant families are included here

The most abundant families detected across PCR replicates with PITS and FITS were found consistently across replicates, but some DNA extracts behaved as outliers in both relative abundance and composition (Figure 6), and several PCR replicates were outliers in their LCBD (Table S7). At a read sampling depth of 5000 and with a five read minimum threshold, LCBD statistics identified 11 such outlier PCR replicates from the YL.1 extract and two from the FO.2 extract for the PITS results, and one outlier replicate from the FO.2 extract in the FITS results (Table S7). Only five of the 17 outliers observed in extrapolated richness (Table S6) are also LCBD outliers.

To explore how read sampling depth and minimum read threshold influence LCBD outliers, we repeated these analyses at all three read sampling depths (1000, 5000, and 10,000 reads) with minimum read thresholds of two, five, or ten reads (Table S7), and performed Chi‐squared tests for significant differences among groups. The number of PCR replicates identified as LCBD outliers increased significantly with higher read sampling depth in the FITS dataset (*p* = 3.861e−15), but not in the PITS dataset (*p* = .25). We found no significant effect of minimum read threshold for either the PITS (*p* = .71) or FITS (*p* = .79) dataset, suggesting that very low abundance taxa represented by fewer than ten reads are not causing outliers. For both the PITS and FITS dataset, we found that DNA extract itself affected the number of observed PCR outliers significantly (both *p* < 2.2e−16).

### 3.5 Variation among PCR replicates in beta diversity distance matrices

**FIGURE 7:**
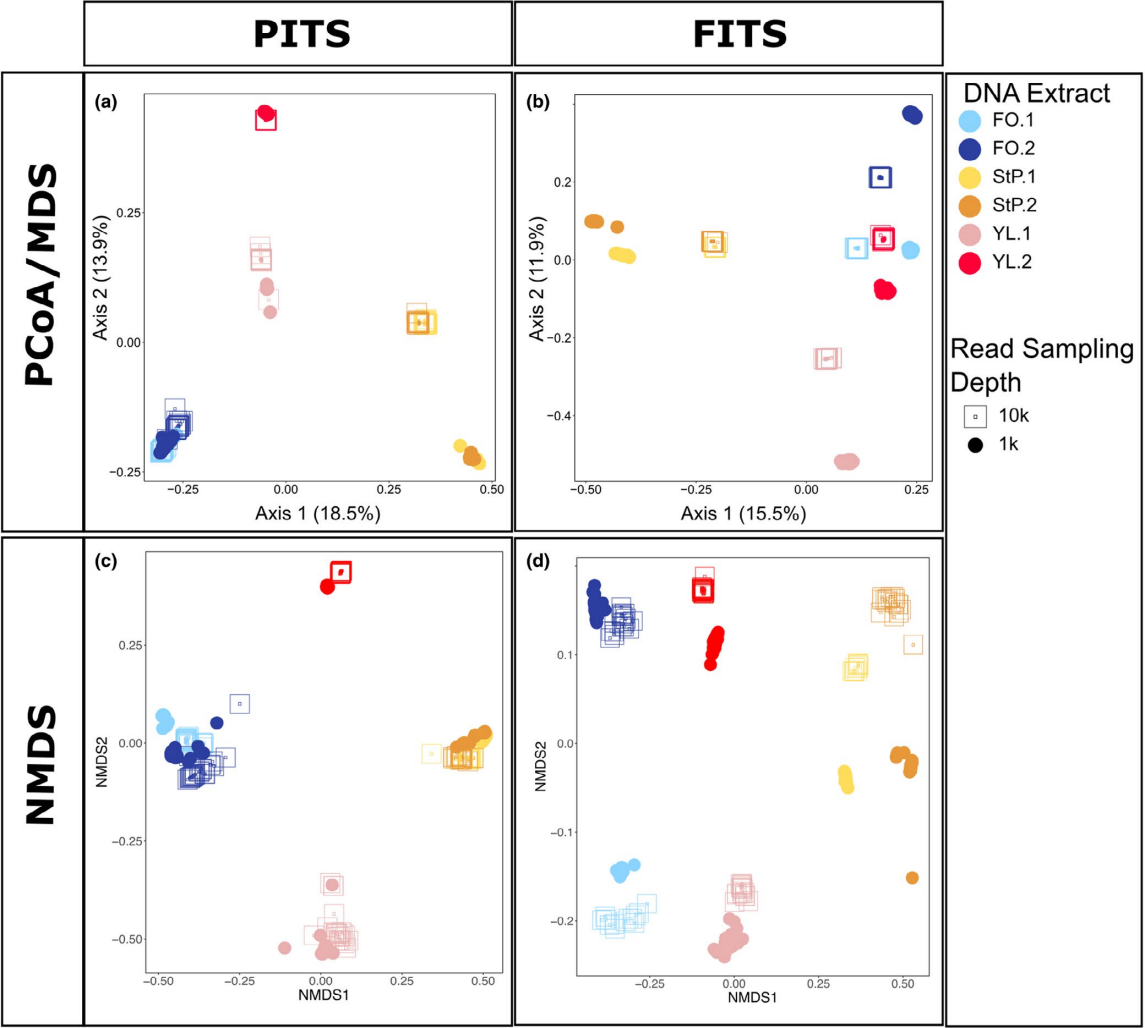
Ordinations of community composition for PITS and FITS datasets. All plots are with a five read minimum threshold. Each point represents one PCR replicate. (a) and (b) PCoA/MDS on Jaccard distances. (c) and (d) NMDS plot on Jaccard distances

We evaluated inter‐ and intra‐extract‐based estimates of beta diversity in ordinations. At both 1000 and 10,000 read sampling depths, and with both PCoA/MDS and NMDS ordinations, PCR replicates in both PITS and FITS results clustered by DNA extract within their sampling depth group, and extracts from the same geographic area sometimes clustered near each other in ordinal space (Figure 7a–d). Clustering by read sampling depth persisted with Bray–Curtis estimates of beta diversity where dissimilarity is weighted by evenness of diversity in addition to richness (Bray & Curtis, 1957; Figure S3). When we increased read sampling depth in PITS PCR replicates was increased from 1000 to 10,000 reads, we saw no difference in NMDS ordination. This may be due to PITS having lower complexity (composition of rare versus common species) compared to FITS. We note that increasing read sampling depth from 1000 to 10,000 reads did increase beta dispersion for the YL2 sample in the PITS dataset (*p*‐value = .0000256; *p* values for all other sites > .5) and for all but one sample in the FITS dataset (FO1 *p* = .000038; FO2 *p* = .000739; YL1 *p* = .000089; YL2 *p* = <.00001; StP1 *p* = <.00001; StP2 *p* not significant = .902146).

Dispersion among PCR replicates is significantly different by metabarcode at a 10,000 read sampling depth (*p* value = .000298), but not at 1000 depth (*p* value = .179292). We plotted the observed richness onto ordinations using RC(M) and saw that most DNA extracts did not have a visible observed richness gradient along an ordination axis (Figures S4 and S5). However, the two taxa that most separate replicates within DNA extract were often low occurrence taxa found in only three or four PCR replicates (Figures S4 and S5). When we plotted these same RC(M) ordinations with Shannon's H measure of alpha diversity, only YL2 in the FITS dataset showed a correlation between alpha diversity and dispersion in beta diversity (Figures S6 and S7). These analyses support that rare taxa, which increase with deeper sequencing depths, destabilize the position of samples in ordinations, and that the extent of this destabilization likely depends on rare taxa, on the community complexity of the metabarcode, and on the chosen ordination method.

## 4 DISCUSSION

Both read sampling depth and the number of PCR replicates significantly affected our measures of alpha diversity of amplifiable molecules within an eDNA extract (Figure 2). We observed stochasticity among PCR replicates in which and how many low abundance taxa were recovered (Figures 4 and 5), as has been shown previously using both simulated and real data (Alberdi et al., 2017; Beentjes et al., 2019; Dopheide et al., 2019; Ficetola et al., 2015; Kelly et al., 2019; Piggott, 2016; Smith & Peay, 2014). When we increased read sampling depth from 1000 to 10,000 reads, observed alpha diversity increased for both the PITS and FITS datasets (Figures 1, 2; Table S5). Shannon and Simpson diversity, which incorporate abundance as well as presence/absence data, increased significantly with all FITS datasets but not for most PITS datasets (Figure 2b, c, e f; Table S5). Given these results, it may not be possible to make generalizable recommendations about replication strategy or sequencing depth. Our experimental data therefore confirm suggestions from simulation studies (Kelly et al., 2019) that diversity measures are sensitive to PCR replication and sequencing depth regardless of metabarcode choice.

We observed the highest variation in alpha diversity both when comparing different sampling depths and between individual replicates at the same sampling depth at the Californian lagoon site (YL.1) (Figures 1e and 2) where water and wind carries and deposits DNA‐containing materials from the surrounding environment. Additionally, while species accumulation curves for each site were still increasing after data from all 24 PCR replicates were added (Figure 3), the timing of saturation of these curves, which we defined as an average increase (after averaging bootstrapped samples) of fewer than one taxon with an added PCR replicate, varied significantly by site, again with the lagoon site the slowest to approach saturation (Table 2; Figure 3). In general, when we either increased the minimum read threshold (the number of reads required for a taxon to be counted as present) or increased the sampling depth of each PCR replicate, fewer replicates were required to saturate the species accumulation curves (Table 2). Together, these results suggest that many unique taxa are rare in sequence abundance in each PCR (as observed in Figure 5). We cannot test, however, whether these rare taxa reflect true rare biodiversity, false positives from polymerase or sequencing error, or low amplification efficiency.

False‐positive taxa, or taxa incorrectly assigned to a particular replicate, can inflate both alpha diversity, and the number of replicates required to saturate species accumulation curves. One source of false positives is index hopping, in which sequences are associated with the wrong indices due to proximate clustering during sequencing (van der Valk et al., 2019). We found no evidence of index hopping among spiral ginger data, suggesting that this was not a major source of noise in our dataset. In addition, we cross‐validated our PITS data table with traditional plant survey data from each site and found no bias in the frequency with which a taxon was observed in PCR replicates compared to its detection with the survey (Text S2; Table S4), suggesting that many low abundance taxa are not false positives. While spiking and cross‐validation offer some evidence of authenticity, identifying false positives remains a challenge in metabarcoding research (Ficetola et al., 2016). Other approaches to detect and remove false positives include establishing minimum read thresholds and/or confirming taxon presence in multiple replicates, but these approaches also remove true positives present at low frequency and impact subsequent analyses (Taberlet et al., 2018; Tsuji et al., 2019). We found, for example, that increasing the minimum read threshold removed some low abundance taxa, reducing alpha diversity and the number of replicates required to saturate the taxon accumulation curve. However, if singletons are removed, as is possible with some software (e.g., BEGUM, Yang et al., 2021), this may artificially reduce biodiversity or complexity estimates. In this study, for example, removing singletons would remove 21–29% of the total taxa identified (Tables S1 and S2). Approaches that generate mock or simulated communities (Ficetola et al., 2015) may help differentiate true and false positives, and Procrustes (Gower, 1975) simulators may help to estimate the likelihood that false positives impact diversity metrics.

While previous work has described the potential impact of rare taxa including false positives on diversity patterns (e.g., Beentjes et al., 2019; Dopheide et al., 2019; Nichols et al., 2017), no consensus has emerged as to how many PCR replicates are necessary to characterize biodiversity within an eDNA extract. Ficetola et al. (2015) estimated from simulated data that eight replicates should be sufficient to detect low abundance taxa, but Dopheide et al. (2019) predicted 10–20 replicates may be required to detect the full biodiversity of some extracts. We observed considerable variation between sites and barcodes in the number of replicates necessary to reach saturation of species accumulation curves (Table 2; Figure 3) and, despite using 24 replicates, most of our rarefaction curves and extrapolated species accumulation curves do not saturate. One explanation for this difference between our and other studies is our use of qPCR to determine the appropriate number of cycles for each sample. Using qPCR in this way makes it less likely that our PCR amplicon pools are overamplified, and therefore more likely that we retain rare taxa (Kelly et al., 2019; Murray et al., 2015). Cumulatively, these results suggest that it may not be possible to exhaustively survey biodiversity using eDNA metabarcoding, in particular for taxa, sites, and metabarcodes with high species richness and large numbers of potentially rare taxa. However, researchers focusing on rare taxa may want to consider an approach like qPCR or baited sequence capture with shotgun libraries to optimize recovery of rare or poorly amplified taxa.

While recovery of low abundance taxa remains challenging, we find that high abundance taxa are consistently recovered, suggesting that low replication may be sufficient to address some biological questions, such as site differentiation. Despite some PCR replicates being LCBD outliers (Table S7), we find strong evidence of consistency among PCR replicates in community composition (Figures 6 and 7) and relative abundance estimates (Figures 6 and S1) within a chosen read sampling depth (i.e., rarefaction depth). However, dispersion among PCR replicates in ordination space differs by ordination metric, metabarcode, and sequencing depth (Figures 7 and S4–S7).

The influence of read sampling depth on beta diversity varies by both metabarcode and site, and can influence dispersion among PCR replicates in ordination space (Figures 7 and S4–S7). Increasing read sampling depth caused replicates from all samples to become less dispersed in Jaccard PCoA/MDS space, but not in NMDS space (Figure 7). Increasing read sampling depth from 1000 to 10,000 reads, for example, made the two St. Paul sites indistinguishable in PCoA/MDS space in the FITS dataset. The St. Paul sites were both dominated by one fungal family (*Hygrophoraceae*), which would have limited the detection of shared rare diversity at low read sampling depths.

Finally, we found that outlier PCRs were more commonly amplified when extracts/sites have high taxonomic diversity. Outlier PCRs were most common in our Younger Lagoon sites, where biodiversity was high, and least common in the Alaskan sites, where biodiversity is lower (Figures 1 and 2; Table S7). When we increased read sampling depth, the frequency of PCR outliers also increased, but only for the FITS datasets (Table S7). This may reflect a combination of the higher number of low abundance taxa recovered by the FITS metabarcode and the low identifiability of sequences amplified by this barcode compared to others due to database limitations. Changing the minimum read threshold, alternatively, did not significantly influence the prevalence of PCR outliers (Table S7), suggesting that the lowest abundance taxa are not determining outlier status. Outliers called by extrapolated richness did not overlap well with outliers called by LCBD, confirming our observation that taxa driving dispersion in beta diversity were low abundance, but not singletons, which inflate alpha diversity estimates (Figures S4–S5). While further work will be necessary to understand the precise cause of outlier PCRs, outliers are only observable (and removable) if more than two PCR replicates are performed. This rationale is often used in experiments that perform three PCR replicates per sample (Taberlet et al., 2018), as this experimental design allows disambiguation between an outlier and nonoutlier replicates.

## 5 CONCLUSION

Here, we investigated the impact of PCR replication, read sampling depth, and minimum read threshold on estimates of alpha and beta diversity from eDNA extracts. At each of our sites and with both metabarcodes, alpha diversity increased with sampling depth and number of PCR replicates and decreased with higher minimum read thresholds. We find that 24 replicates, a number higher than the standard recommendations in the field, were too few to survey the complexity of taxa that are amplifiable using either metabarcode, suggesting that the ubiquitous nature of rare taxa and extract unevenness may make exhaustively surveying biodiversity from eDNA extracts impossible. We also find that, while beta diversity is stable among PCR replicates rarefied to the same number of reads, differences in read sampling depth led to shifts in ordination space. These shifts can span greater distances than two samples collected from different biomes (Figure 7). Future research using simulations with natural community biodiversity and with different primers and laboratory assays will be key to determining the extent that we can estimate and discriminate false negatives and positives, as well as estimate the volatility of alpha diversity and beta diversity within extract or among extract or sites. We imagine that simulations testing the robustness of patterns in multiple analyses, followed by empirical observation of community composition in natural community positive controls, or wider use of mixes such as ZymoBIOMICS Microbial Community DNA Standard II (Zymo Research), will help diagnose the vulnerabilities of different methodological choices to biases from rare taxa.

Together, these results reiterate the importance of considering physical and ecological settings as well as the targeted taxa and metabarcode choice as part of experimental design (Andersen et al., 2011; Ficetola et al., 2015). Experimental parameters not investigated here also affect biodiversity estimates from eDNA samples, including DNA extraction method (Deiner et al., 2018; Dopheide et al., 2019; Piggott, 2016), the amount of soil processed (Dopheide et al., 2019), and metabarcode choice (Alberdi et al., 2017; Duke & Burton, 2020). Nonetheless, this work contributes to understanding of the complexity of eDNA research and underscores the power of simplified experiments that hold some parameters constant while allowing others to vary to facilitate development of experimental strategies that maximize the impact of eDNA.

## CONFLICT OF INTEREST

The authors have no conflicts of interest to report.

## AUTHOR CONTRIBUTION

**Sabrina Shirazi:** Conceptualization (equal); data curation (lead); formal analysis (lead); investigation (lead); methodology (lead); visualization (lead); writing—original draft (lead); writing—review and editing (equal). **Rachel S Meyer:** Data curation (supporting); formal analysis (supporting); investigation (supporting); supervision (supporting); writing—review and editing (equal). **Beth Shapiro:** Conceptualization (equal); formal analysis (supporting); funding acquisition (lead); supervision (supporting); writing—review and editing (equal).

## Supporting information

Fig S1‐S7

Table S1

Table S2

Table S3

Table S4

Table S5

Table S6

Table S7

Supinfo S1

Supinfo S2

## Data Availability

Raw sequencing data are available on the NCBI SRA (BioProject PRJNA719103).
