# Supplementary material for: Revisiting the effect of PCR replication and sequencing depth on biodiversity metrics in environmental DNA metabarcoding
Source: Ecol Evol. 2021 Oct 22;11(22):15766–79. doi: 10.1002/ece3.8239 (PMC8601883; doi:10.1002/ece3.8239)
Supplement: Supplementary file 1 — Fig S1‐S7 [file ECE3-11-15766-s003.pdf]

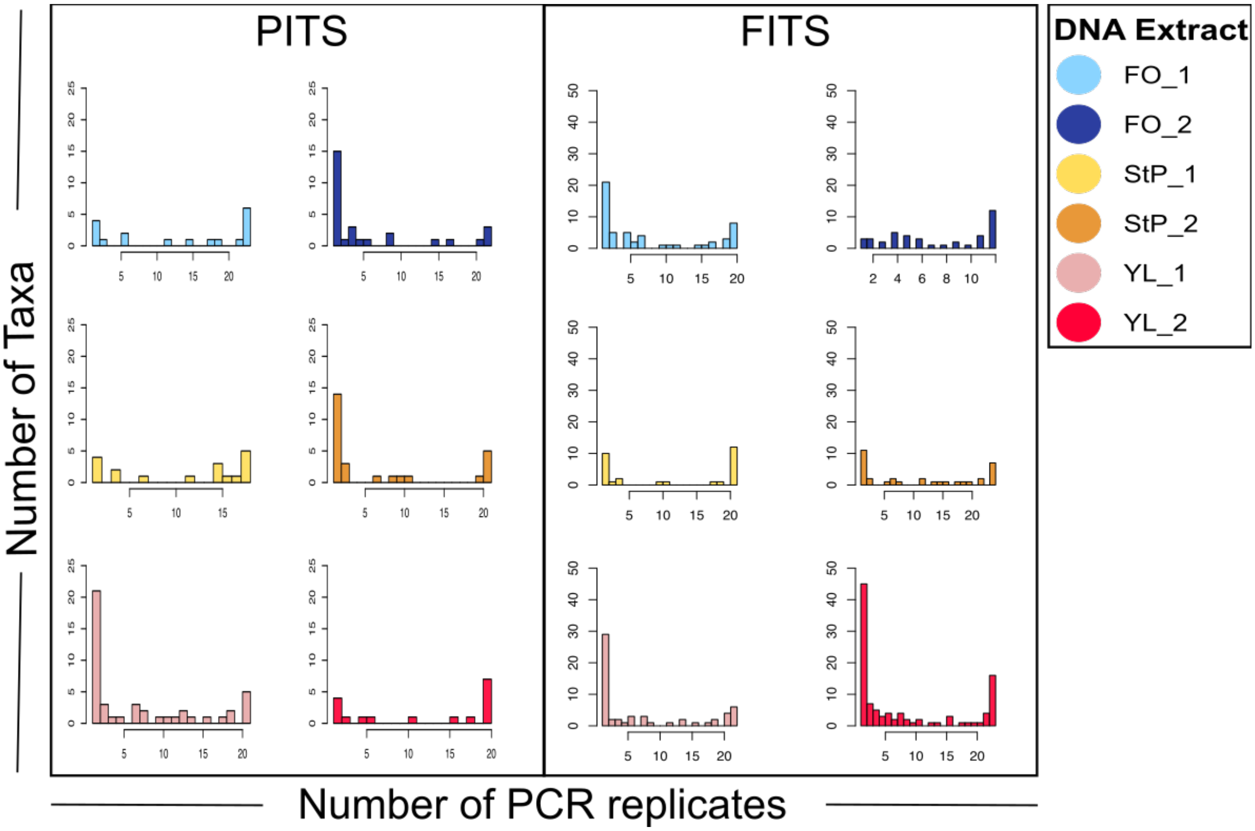

S. Fig1. Histograms describing the frequency of individual taxa detected across PCR replicates, each sampled to a read depth of 5000 reads and using a minimum read cutoff of ten, out of the total 24 replicates.

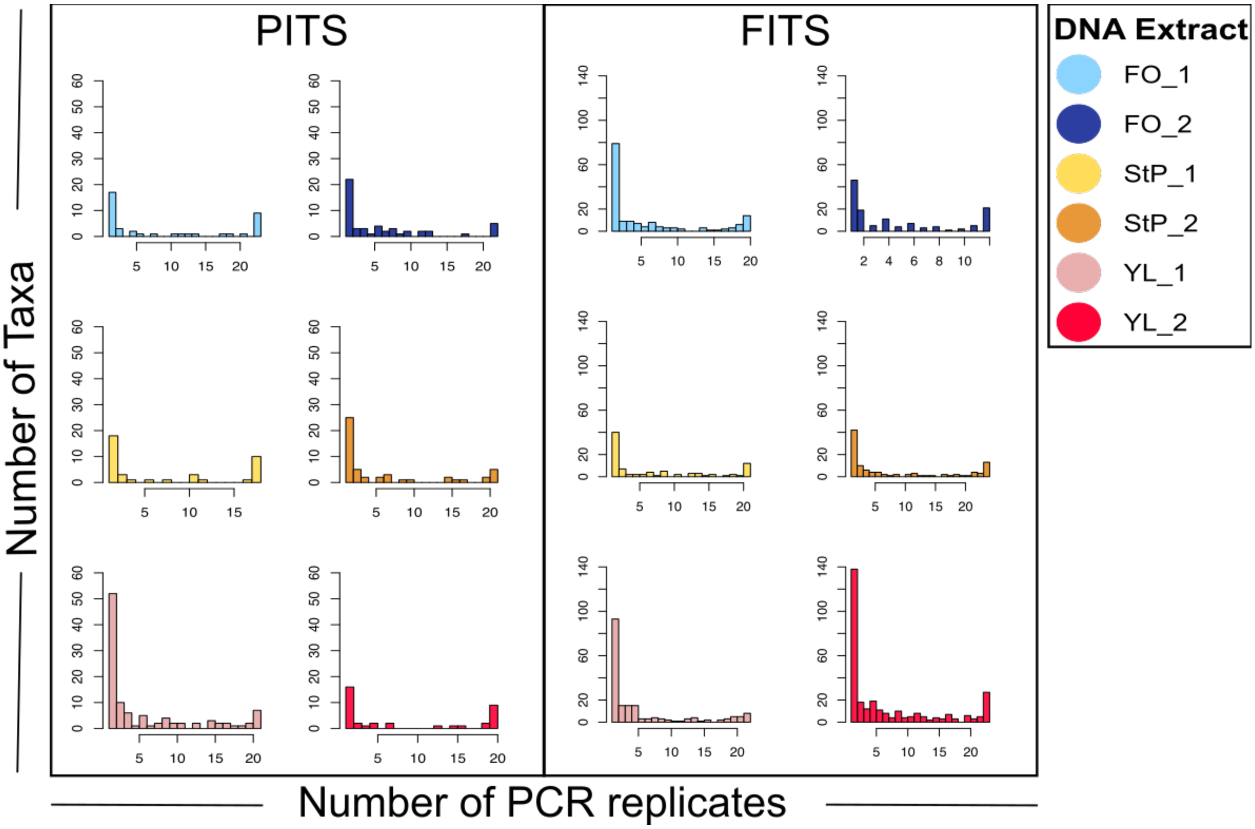

S. Fig2. Histograms describing the frequency of individual taxa detected across PCR replicates, each sampled to a read depth of 5000 reads and using a minimum read cutoff of two, out of the total 24 replicates.

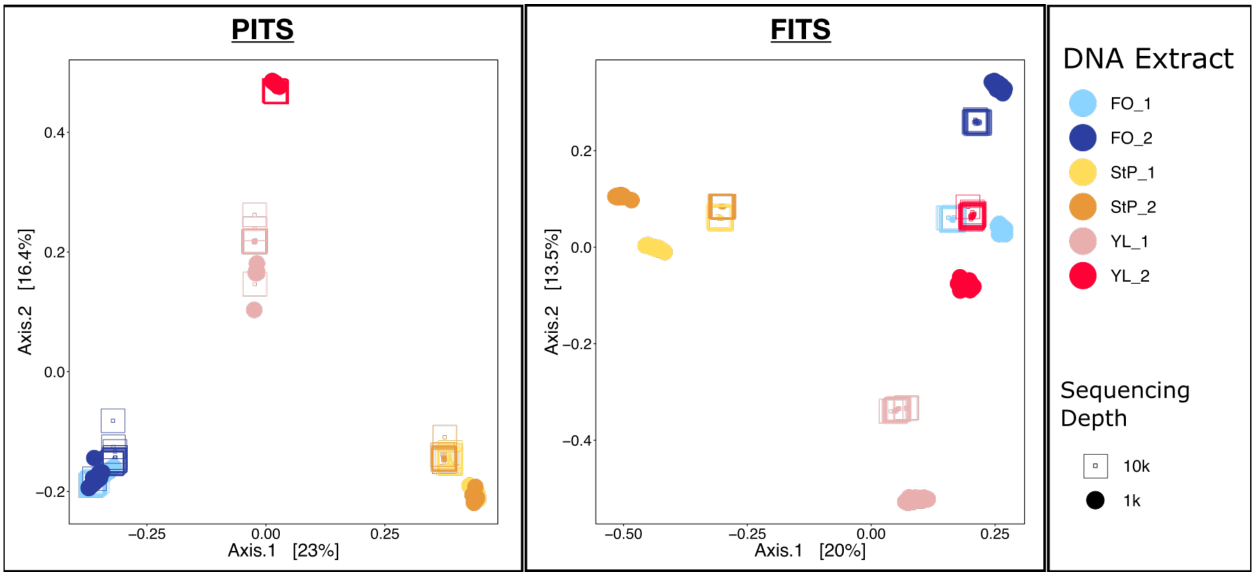

S. Fig3. PCoA of PITS and FITS datasets at sequencing depths 1k and 10k using a Bray-Curtis measure of β diversity

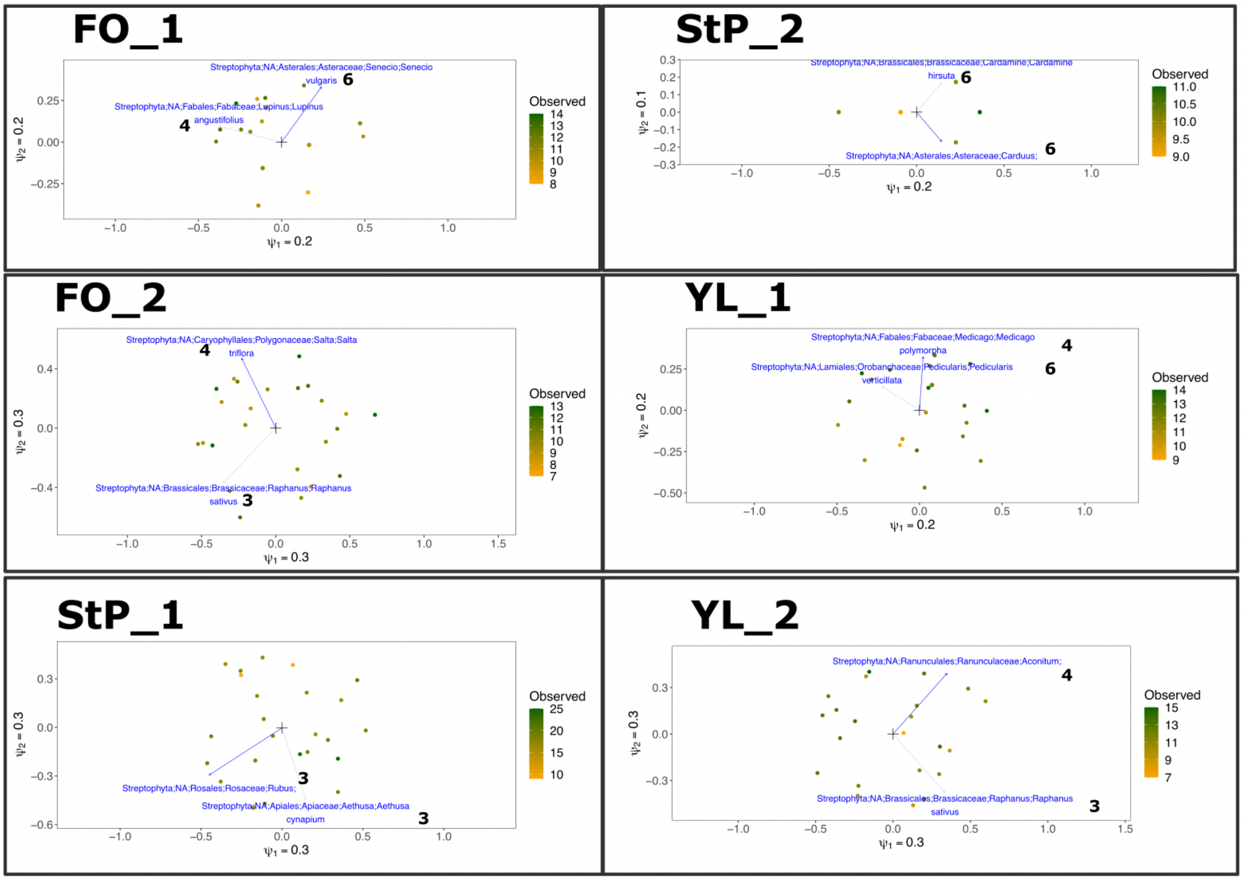

S. Fig4. RC(M) plots for PITS dataset rarefied to 5000 reads and a minimum 5 read cutoff with samples colored by their Observed richness values and the top 2 taxa separating samples shown as vectors. Bold numbers are the number of PCR replicates the taxon is found in, showing most of these top taxa only occur in a few replicates.

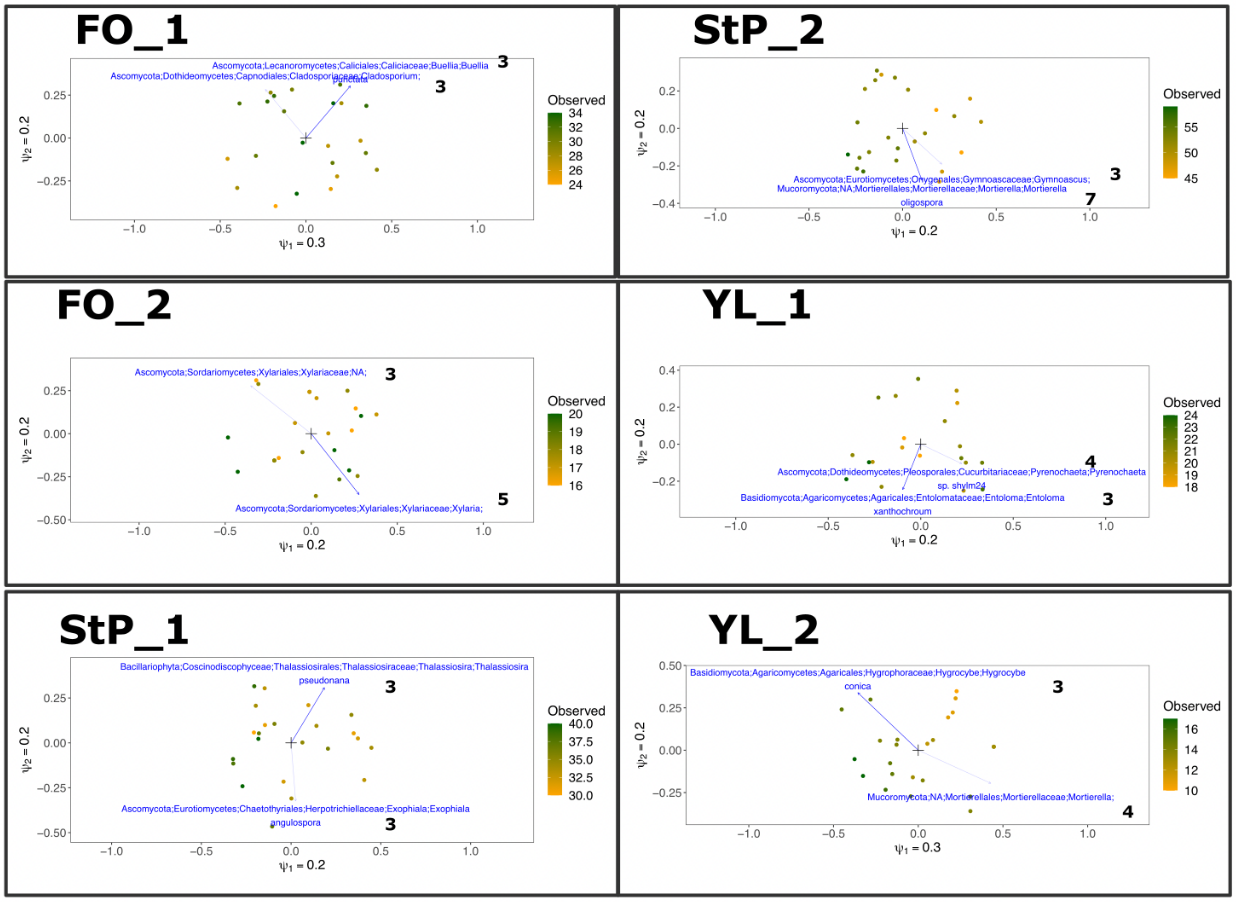

S. Fig5. RC(M) plots for FITS dataset rarefied to 5000 reads and a minimum 5 read cutoff with samples colored by their Observed richness values and the top 2 taxa separating samples shown as vectors. Bold numbers are the number of PCR replicates the taxon is found in, showing most of these top taxa only occur in a few replicates.

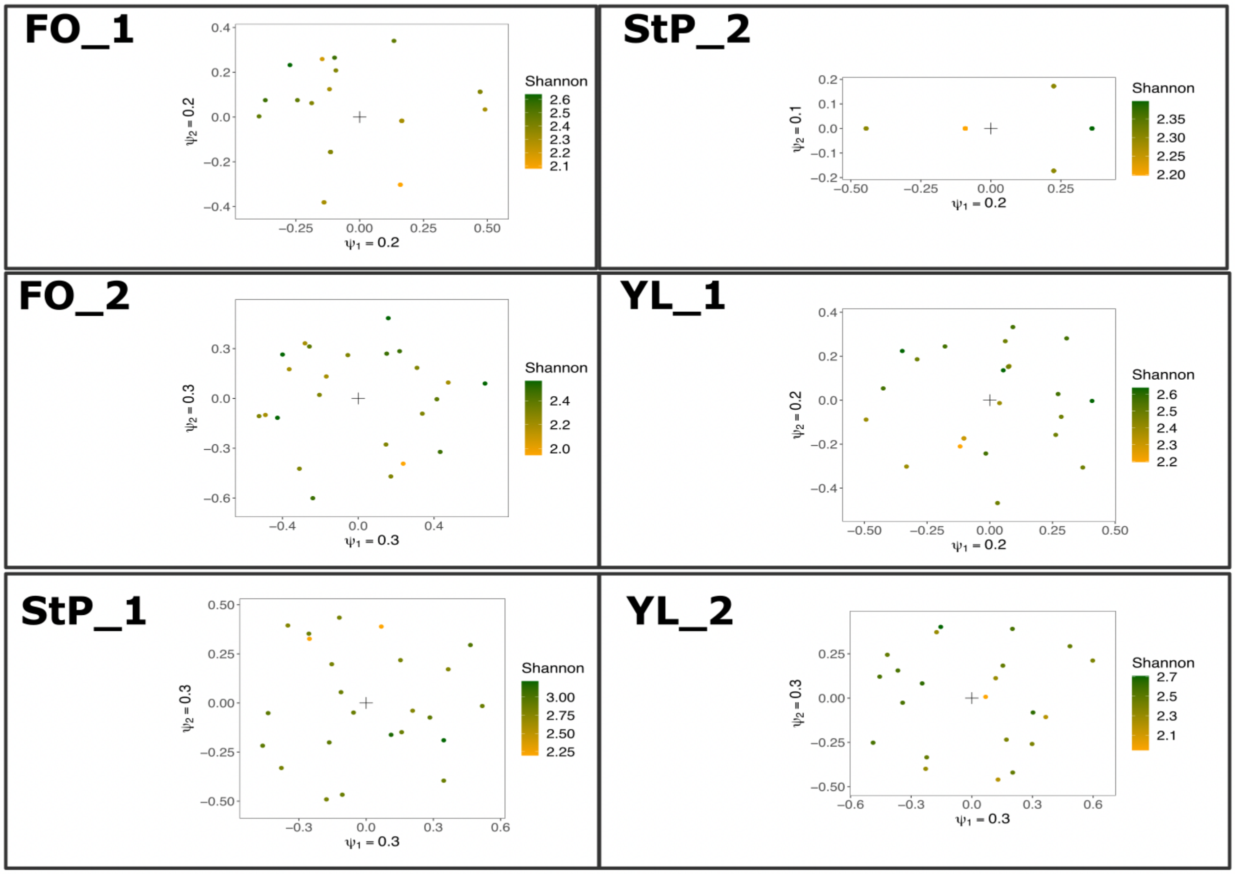

S. Fig6. RC(M) plots for PITS dataset rarefied to 5000 reads and a minimum 5 read cutoff with samples colored by their Shannon's H richness values.

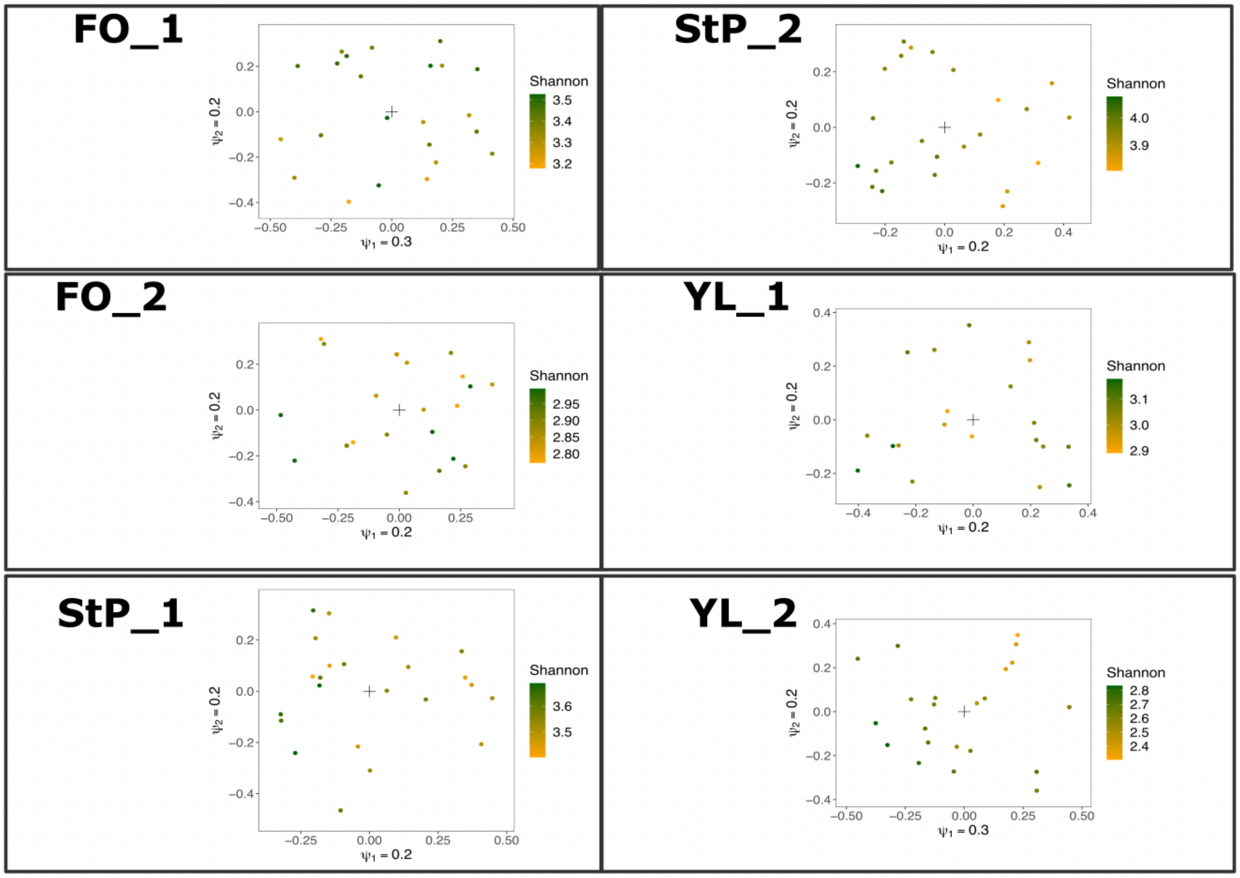

S. Fig7. RC(M) plots for FITS dataset rarefied to 5000 reads and a minimum 5 read cutoff with samples colored by their Shannon's H richness values.
