# Supplementary material for: Revisiting the effect of PCR replication and sequencing depth on biodiversity metrics in environmental DNA metabarcoding
Source: Ecol Evol. 2021 Oct 22;11(22):15766–79. doi: 10.1002/ece3.8239 (PMC8601883; doi:10.1002/ece3.8239)
Supplement: Supplementary file 9 — Supinfo S1 [file ECE3-11-15766-s009.docx]

Supplement 1.

*Anacapa* is a metabarcoding data processing pipeline that enables simultaneous data processing of multiplexed barcodes and libraries. Within *Anacapa*, we trimmed the TruSeq adapters using *cutadapt* (Martin, 2011), removed bases with Q-scores below 35 with the *FastX-Toolkit* (Gordon and Hannon, 2010), and then trimmed, again using cutadapt. We trimmed the first 5 bases on the 5’ end of the forward read and the first 10 bases of the 5’ end of the reverse read for the PITS data set. We trimmed 40 bases off the 5’ end of the forward read and the first 50 bases of the 5’ end of the reverse read for the FITS data set. We used *dada2* (Callahan et al. 2016) to merge the forward and reverse reads, remove chimeric sequences, and identify amplicon sequence variants (ASVs). We performed merged, unmerged paired, and single read ASV assignment to taxa via global and local alignment using *Bowtie2* (Langmead and Salzberg, 2012) to reference CRUX databases released in Curd et al. 2019. The top 100 hits of the *Bowtie2* alignment to reference were bootstrapped with *BLCA* (Gao et al, 2017) to assign each ASV to a taxon and provide uncertainty estimates. We used a 60% bootstrap confidence threshold of taxonomic assignment, as suggested in the *Anacapa* documentation (Curd et al, 2019). *Anacapa* output two taxonomy tables (one per amplicon) formatted as matrices of the number of reads from each PCR replicate assigned to a given taxa.

As a recently published database generation tool, CRUX databases are created using a multi-step process that begins with an EcoPCR using primer sequences and a target amplicon length to pull out e-amplicons from the EMBL nucleotide database (Stoesser et al., 2002). This creates a set of seed marker sequences that are used to query the NCBI nr/nt database twice, first accepting only full length reads, then accepting reads at 70% full length, retrieving up to 10,000 sequences per seed sequence query. This latter step allows us to find and include in the database sequence entries that do not include the primer sites and that are not sequenced across the entire barcode region. The resulting data are dereplicated by retaining only unique sequences, and any taxon labeled as ‘environmental sample’ is removed. Closed reference databases such as CRUX databases are commonly used in metabarcoding because they are more rapidly queried compared to open databases and can be quality curated. Prior to generating the eDNA results used in this study, we compared CRUX databases published in Curd et al., (2019) to the UNITE (Nilsson et al., 2018) and found CRUX databases identified more taxa when applied to our eDNA data.

**References:**

Callahan, B. J., McMurdie, P. J., Rosen, M. J., Han, A. W., Johnson, A. J. A., & Holmes, S. P. (2016). DADA2: High-resolution sample inference from Illumina amplicon data. *Nature Methods*, 13(7), 581–583.<https://doi.org/10.1038/nmeth.3869>

*Gordon, A.*, & *Hannon, G. J.* (*2010*). Fastx‐toolkit. FASTQ/A Short‐Reads Preprocessing Tools (Unpublished). [*Http://Hannonlab.Cshl.,Edu/Fastx_toolkit,5*](about:blank).

Langmead, B., & Salzberg, S. L. (2012). Fast gapped-read alignment with Bowtie 2. *Nature Methods*, 9(4), 357–359. https://doi.org/10.1038/nmeth.1923

Martin, M. (2011). Cutadapt removes adapter sequences from high-throughput sequencing reads. EMBnet.Journal, 17(1), 10. https://doi.org/10.14806/ej.17.1.200

Stoesser, G., Baker, W., van den Broek, A., Camon, E., Garcia-Pastor, M., Kanz, C., Kulikova, T., Leinonen, R., Lin, Q., Lombard, V. and Lopez, R., 2002. The EMBL nucleotide sequence database. Nucleic acids research, 30(1), pp.21-26.
