# Supplementary material for: Revisiting the effect of PCR replication and sequencing depth on biodiversity metrics in environmental DNA metabarcoding
Source: Ecol Evol. 2021 Oct 22;11(22):15766–79. doi: 10.1002/ece3.8239 (PMC8601883; doi:10.1002/ece3.8239)
Supplement: Supplementary file 10 — Supinfo S2 [file ECE3-11-15766-s007.docx]

Supplement 2.

As there is no standard way to differentiate false and true positives, we used cross-validation with traditional observation data, similarly to recent studies (McElroy et al., 2020; Lin et al., 2021), to characterize whether taxa occurring at low frequency were likely to be false positives. However, due to the enormous uncharacterized biodiversity within Fungi, we employed this cross-validation with PITS results only. We found 31 of the 161 genera from the PITS dataset were from Chlorophyta and only two of these were cross-validated in traditional observation datasets (Table S8). We attributed this low overlap to observation bias and chose not to consider Chlorophyta further. In contrast, 100 of 129 Streptophyta genera were cross-validated (Table S8). Those 29 other genera occurred in nine orders, eight of which contained cross-validated genera (Klebsormidiales was the exception). We hypothesized that if these 29 genera represented some false positives they would be found at lower frequency in PCR replicates than cross-validated genera, and observed that the 29 genera had significantly lower frequency of detection than the 100 cross-validated Streptophyta genera based on a one-tailed t-test (*p=*0.038, t-value=-1.79).

To further assess putative false positives, we examined the frequency of congener species across PCR replicates. We observed that 42% of recovered PITS genera have multiple congener species in our dataset. When we tested whether taxa in a single replicate were congeners with taxa found in other replicates, we found that taxa present in a single replicate (group 1, M=5.35, ss=4853.41) were no more likely to belong to congener groups than taxa in multiple PCR replicates (group 2, M=4.98, ss=9063.94; t=0.48252, *p*=0.31487), indicating that these congeneric taxa, whether true positives or not, are not overrepresented as singleton observations in PCR replicates. We concluded that our metabarcoding data represent a mixture of low frequency taxa that are largely true positives but contain some false positives, as is common and expected in metabarcoding, and therefore that evaluating similarity among our PCR replicates has the potential power to inform decisions about further filtering based on taxa shared among replicates.

References:

Lin, M., Simons, A. L., Harrigan, R. J., Curd, E. E., Schneider, F. D., Ruiz‐Ramos, D. V., Gold, Z., Osborne, M. G., Shirazi, S., Schweizer, T. M., Moore, T. N., Fox, E. A., Turba, R., Garcia‐Vedrenne, A. E., Helman, S. K., Rutledge, K., Mejia, M. P., Marwayana, O., Munguia Ramos, M. N., … Meyer, R. S. (2021). Landscape analyses USING edna metabarcoding and Earth observation predict community biodiversity in California. *Ecological Applications*. https://doi.org/10.1002/eap.2379

McElroy, M.E., Dressler, T.L., Titcomb, G.C., Wilson, E.A., Deiner, K., Dudley, T.L., Eliason, E.J., Evans, N.T., Gaines, S.D., Lafferty, K.D. and Lamberti, G.A.. (2020). Calibrating environmental DNA metabarcoding to conventional surveys for measuring fish species richness. *Frontiers in Ecology and Evolution*, *8*, p.276.
